# Recorded Offending Trajectories Preceding Different Sanction Types: A Register-Based Study on the Ladder of Punishments

**DOI:** 10.1007/s40865-026-00326-8

**Published:** 2026-10-15

**Authors:** Aaro Beuker, Karoliina Suonpää, Antti Latvala, Mikko Aaltonen, Pekka Martikainen

**Affiliations:** 1 Institute of Criminology and Legal Policy, University of Helsinki, Helsinki, Finland; 2 Law School, University of Eastern Finland, Joensuu, Finland; 3 Helsinki Institute for Demography & Population Health, University of Helsinki, Helsinki, Finland

**Keywords:** Criminal sanctions, Register data, Criminal careers, Sanctioning, Longitudinal research

## Abstract

This study examines officially recorded offending trajectories leading to different types of criminal sanctions in the Finnish justice system. We focus on the most common sanctions in terms of quantity, with emphasis on suspended imprisonment, community service, and imprisonment. The choice of sanction type is not solely based on the severity of the crime committed, but also on the offender’s criminal history and assessed capability to comply with the requirements of non-custodial sanctions. Individual sentences and their justifications are always the result of judges’ discretion, but a large-scale analysis of the sanction trajectories and recorded offenses preceding different sanction types provides important information about the functioning of the criminal justice system. By using detailed, whole population register data from birth cohorts 1980–1990 (*N* = 714,607), we analyze recorded offending trajectories preceding the offenders’ first sanction type of each kind, that is, the entry to different stages of the punishment ladder. The results show that while first suspended imprisonments are not preceded by extensive criminal histories, they are rarely the first criminal justice contact. Individuals sentenced to community service or imprisonment for the first time have accumulated a large number of prior criminal convictions and crimes before receiving these sanction types, with a median of 9 and 13 prior sanctions, respectively.

**Supplementary Information:**

The online version contains supplementary material available at https://doi.org/10.1007/s40865-026-00326-8.

## Introduction

The principle of proportionality is a central element in evaluating the severity of a punishment (Chiao, 2018; Lappi-Seppälä, 2016; Wenander, 2020). When determining a just punishment that corresponds to the offense, proportionality is reflected in both the choice of sanction type and the extent of the sentence (Lappi-Seppälä, 2022). Consequently, sanction types have a hierarchy of severity, with imprisonment typically at the highest level and fines at the lowest, resulting in a ladder model of punishment (Hinkkanen & Lappi-Seppälä, 2011). However, the criminal histories preceding different sanction types have not been thoroughly examined using data that allow for the examination of individual-level offending trajectories. Investigating this provides insights that enable evaluation of how the proportionality principle is reflected in the justice system; an important topic, since persistent offending is considered an aggravating element when determining a sentence (Criminal Code of Finland Chap. 6, Sect.  5).

Since the population of persons serving a criminal sentence in Western societies is primarily composed of individuals undergoing non-custodial sentences, most offenders are positioned at the lower end of the punishment ladder. In the US, a country with a high incarceration rate in the Western context, 56% of the correctional population was serving probation at the end of 2023, corresponding to 3.1 million convicts (Gann & Kaeble, 2025). In Europe, non-custodial sanctions such as suspended imprisonment and community service have become increasingly common alternatives to incarceration. Although there are differences in utilization rates, most European countries favor non-custodial alternatives to imprisonment in minor and middle-range offenses, with fines and suspended imprisonment considered the main sanction types (Rodrigues et al., 2022).

While penal policies seem to be weighted toward non-custodial sanctions – especially for first time offenders – the crime careers preceding these sentences remain understudied. Countries typically report aggregate general penal statistics, for example yearly amounts of sanction types imposed with related crime type and demographic data (e.g., Schmitt et al., 2025). However, the information provided does not address what types of offenders receive these sanctions. A study by Tahamont et al. (2015) has contributed to this topic by analyzing offending trajectories of first-time prisoners in New York State, concluding that most individuals incarcerated for the first time had had multiple prior contacts with the criminal justice system before being sentenced to imprisonment. Since the publication of this study, little follow-up research has emerged.

Consequently, there is a lack of research on offending trajectories preceding sanction types other than imprisonment, which, considering the significance of non-custodial sanctions, we identify as a major shortcoming. This is especially the case in the Nordic context, in which the use of non-custodial sanctions is extensive. Low incarceration rates with an emphasis on rehabilitative measures – characteristics of Nordic penal exceptionalism (Lappi-Seppälä 2012; Pratt 2007a, b) – highlight the need for further research on offending trajectories preceding different sanction types. Studies analyzing the effectiveness of non-custodial sanctions have increased in recent years (see for example: Andersen, 2024; Andersen & Telle, 2022; Al Weswasi & Bäckman, 2025; Huttunen et al., 2020), but these studies do not provide detailed information on the thresholds for the imposition of these sanction types, particularly in relation to the criminal records and officially recorded crimes of the individuals receiving criminal sanctions. In addition to impact evaluations, an understanding of the pathways to different sanction types is needed.

In this study, we apply approaches from life-course criminology as we investigate several key questions regarding the timing and structure of offending prior to the first sanction type of each kind, with a focus on suspended imprisonment, community service, and imprisonment. By using official register data from Finnish district courts, we analyze the officially recorded crimes and sentences preceding sanction types that differ in their severity. This provides an overview of the general characteristics of different stages of the punishment ladder, regarding the gradual escalation in severity of punitive measures. Tracing backwards from the first instance of each sanction type imposed on individuals, we provide new evidence on how the ladder of punishments is realized in the present-day criminal justice system.

### Officially Recorded Offending Trajectories

The study of offending trajectories refers to the analysis of criminal behavior over time. A life-course perspective of offending trajectories typically examines how life circumstances such as age (Loeber & Farrington, 2014; Steffensmeier et al., 2025), developmental challenges (Ångström et al., 2024; Jolliffe et al., 2017) or life events (Ramakers et al., 2020) are associated with criminal activity, bringing attention to the timing of these factors in relation to offending. However, with official sentencing data, offending trajectories can also be analyzed as sanction trajectories, focusing on the types and sequences of sanctions imposed on offenders. The analysis of officially recorded offending and sanction trajectories can provide valuable information about the functioning of the criminal justice system. This approach was applied by Tahamont et al. (2015), who used detailed register data from New York State to analyze offending trajectories preceding imprisonments. Their study concluded that while most first-time prisoners had multiple prior contacts with the criminal justice system, a quarter of the prisoners had not been arrested before the arrest that led to their first prison admission. According to these findings, interventive measures could be directed at known offenders with high offending rates, but there is little that the justice system can do in the case of previously unknown offenders.

The longitudinal patterns of criminal activity have also been studied from the perspective of the criminal career concept, applying similar methodological strategies to life-course criminology but focusing more on measuring criminal careers and identifying offender groups who engage in differing levels of criminal activity (Sullivan & Piquero, 2017).The criminal career approach has been adopted in studies which have drawn upon longitudinal register data from administrative sources. It has been shown that a small number of offenders is responsible for a large proportion of overall crime (Falk et al., 2014; Sivertsson et al., 2021); but at the same time, a significant proportion of population has received a criminal sanction at least once in their life (Ångström et al., 2024; Sivertsson et al., 2021; Skardhamar, 2014). These findings indicate that while crime and receiving a criminal sanction can be seen as relatively common, criminal acts are still disproportionately concentrated within a small fraction of the population.

Drawing on the work of Tahamont et al. (2015) and register-based crime career studies, we extend the research to non-custodial sentences using official register data from Finnish district courts. This research is essential for understanding the differences in offending trajectories and criminal backgrounds among individuals entering the different stages of the penal system. First, if patterns are detected, it offers insight into potential opportunities for interventive measures. Second, it provides information about the functioning of the legal system, offering a broad overview of sanctions imposed by decision-making authorities. 

### The Judicial Context

The Finnish criminal justice system uses several sanction types that vary in their penal severity. Courts impose fines, suspended imprisonment, community service, and imprisonment (Lappi-Seppälä, 2022); these can be considered the main sanction types if measured by the number of sentences imposed in courts (Kuusisto Mäkelä, 2023). In addition to court-ordered sentences, police officers and prosecutors may issue minor fines, which are issued for crimes (e.g. traffic crimes and thefts) that do not need to be evaluated by the court and that the offender does not contest (Lappi-Seppälä, 2022).

In sentencing, the sanction type is determined based on the severity of the offense and the offender’s criminal history, meaning the more severe end of the sentencing scale is applied to those who commit serious offenses and/or have a history of repeated offending (Lappi-Seppälä, 2022). Minor offenses and many middle-range offenses are punished by fines (e.g., endangerment of traffic safety, petty theft, drug use offenses), while more severe non-custodial sanctions, suspended imprisonment in particular, are typical punishments for some of the middle-range and more severe offenses (e.g., assault, fraud, aggravated DUI) (Lappi-Seppälä, 2019).

The emerging importance of non-custodial sanctions is illustrated by recent studies conducted using Finnish register data. These studies have found that the population-adjusted rate of prison convictions, average daily number of prisoners, and proportion of persons sentenced to prison by birth cohort have all decreased in the past decade (Aaltonen et al., 2024). Furthermore, the share of custodial sentences among all convictions has decreased, while the proportion of non-custodial sanctions, mainly suspended imprisonments, has increased (Kuusisto Mäkelä, 2023). Most imprisonments eligible for suspended imprisonment are imposed as such, especially for first time offenders (Kemppinen, 2024; Tyni et al., 2024). Community service, introduced in 1995, played a notable role in the declining prison rate (Aaltonen et al., 2024). These findings and trends concerning sentencing structure highlight the need for studies regarding non-custodial sanctions.

Research on criminal sanctions has mainly focused on prisoners, while other conviction types have received less attention. Finland offers an advantageous empirical context to address this issue, as the sentences imposed are concentrated on non-custodial sanctions. Furthermore, the high-quality register data from district courts and authorities include extensive records of different sanctions from lesser fines to prison sentences with both the pronouncement dates of the sentences and the dates of offenses available, enabling us to analyze the timing and order of these sanctions imposed on individuals. 

### Choice of Sanction Type

As stated before, suspended imprisonment and community service are the main alternatives to short-term imprisonments and can be considered if certain criteria are met. Community service is seen as a more severe sanction than suspended imprisonment as it entails a higher degree of control over the convict. To ensure that community service is only used as an alternative to imprisonment, the court first makes a decision between suspended and mandatory imprisonment (Lappi-Seppälä, 2022). Community service can be imposed in lieu of short-term imprisonment if the length of the imprisonment is eight months or less.

While principles and criteria for sentencing are included in the Criminal Code, the courts still have extensive freedom in choosing the most suitable sanction type (Hinkkanen & Lappi-Seppälä, 2011). Previous criminality on the part of the offender is stated to be a potential aggravating factor (Criminal Code of Finland Chap. 6, Sect.  5), but what this means in practice, i.e., when determining a sentence, is not specified in the Criminal Code. The Finnish justice system does not have formal sentencing guidelines (Hinkkanen & Lappi-Seppälä, 2011), meaning there is no binding criterion regarding persistent offending as an aggravating factor. Repeated offending as an aggravating factor has instead been evaluated in the Supreme Court, whose decisions guide how sentences are determined in the lower courts. Multiple similar minor crimes (Supreme Court of Finland 2018:44) or fewer severe crimes of similar nature (Supreme Court of Finland 2019:21), as well as an extensive criminal history of multiple different offenses (Supreme Court of Finland 2011:66) have all been deemed grounds to aggravate the sentence. 

### Suspended Imprisonment

Suspended imprisonment means that imprisonment is postponed and enforced only if the convict reoffends during their probationary period (Criminal Code of Finland Chap. 6, Sect.  9). Suspended imprisonment can be considered if the maximum sentence does not exceed two years of imprisonment. If the convict commits a serious crime during the probation period, the remaining length of the imprisonment sentenced as suspended is enforced. Suspended imprisonments can be converted to imprisonment only if the convict commits another crime punishable by imprisonment during the probation period (Lappi-Seppälä, 2022).

Suspended imprisonment is the most widely used non-custodial sentence in the Finnish justice system. In 2022, 66% of imprisonments with a maximum length of two years were imposed as suspended (Kuusisto Mäkelä, 2023). For first-time offenders the proportion is even higher (Kemppinen, 2024). The proportion of imprisonments imposed as suspended has grown from one-third in 1970 to two-thirds in 2022 (Kemppinen, 2024; Kuusisto Mäkelä, 2023).

The decision between unsuspended and suspended imprisonment is determined by the length of the sentence, the severity of the crime committed and the offender’s criminal history (Criminal Code of Finland Chap. 6, Sect.  9). As the length of the imprisonment being considered approaches two years, the conditions supporting the choice of suspended imprisonment should become more demanding, as explicitly stated in a decision by the Finnish Supreme Court (Supreme Court of Finland, 2010:18). However, recent research on first-time offenders suggests that the shift towards imposing imprisonments as unsuspended occurs after the length of sentence exceeds a year and a half, indicating that before this cutoff, the length of the sentence is the driving factor in determining the sanction type (Kemppinen, 2024). 

### Community Service

Community service was first introduced in 1991 as a localized trial, which was expanded to a nationwide trial in April 1994. Although it did not become an official part of the criminal sanctions system until 1997, it was already in nationwide use by 1995 (Aaltonen et al., 2024; Leinonen et al., 2019). Imprisonment can be imposed as community service if the length of the prison sentence is eight months or less, and if the offender is deemed suitable and motivated to comply with the sentence (Criminal Code of Finland Chap. 6, Sect.  11). Community service consists of unpaid work in the public sector or a non-profit organization, carried out in the offender’s leisure time. The amount of work required varies from 14 to 240 h and is dependent on the length of the imprisonment, with one day of prison equaling one hour of community service (Matikkala, 2021).

It is notable that the number of persons sentenced to community service has decreased substantially since its full implementation in 1997 (Leinonen et al., 2019). This drop has been attributed to a decrease in the amount of imprisonments convertible to community service, further explained by a decrease in the number of convictions concerning aggravated driving under the influence (Aaltonen et al., 2024). It has also been argued that the sentencing practice for driving under the influence has shifted from community service to suspended imprisonment (Leinonen et al., 2019). 

### Imprisonment

Imprisonments are imposed only for the most severe offenses and repeated offenders (Hinkkanen & Lappi-Seppälä, 2011). In 2024, Finland’s incarceration rate was 51 prisoners per 100,000 residents, a rate that has remained relatively stable in recent years and is clearly below the European average of 177 (Fair & Walmsley, 2024). The prison population in Finland is dominated by recidivists, with a large proportion of persons sentenced to prison committing a new prison-worthy crime within three years of release (Tyni, 2020).

In 2022, the average length of imprisonment was 13 months (Kuusisto Mäkelä, 2023). When measured by the number of sentences imposed, the most common crime types leading to imprisonment are DUIs, thefts and drug-related crimes, whereas drug-related and violent crimes account for the greatest amount of prison time sentenced (Kuusisto Mäkelä, 2023). In recent years, the prison population has grown (Prison and Probation Service Finland, 2025; Tyni, 2020) and become more disadvantaged than before (Aaltonen et al., 2024).

### The Current Study

While prior studies have focused on prisoners (Aaltonen et al., 2024; Ramakers et al., 2020, 2024), the offending trajectories and criminal histories of persons sentenced to non-custodial alternatives remain unclear. Criminal histories are taken into account when the court considers which sanction type to impose, meaning the choice of sanction type is to some extent impacted by the severity of the offender’s criminal record.

By using detailed sentencing data from district courts, we were able to analyze the timing of and trends in offending prior to first sanction type of each kind, referred to as the index sanction. While it is known that repeated offenders are largely represented in the most severe sanction types (Kuusisto Mäkelä, 2023; Prison and Probation Service Finland, 2025), the offending trajectories and crime types leading to first sanction type of each kind have not been analyzed in detail before. Research on the first-time recipients is crucial, as previous severe convictions can constitute an obstacle to receiving more lenient sanction types (Criminal Code of Finland Chap. 6, Sect.  11), creating a potential bias if researching criminal histories preceding different sanction types without the focus on first sanction types of each kind.

We compared the criminal histories of persons sentenced to different sanction types by including all criminal convictions of persons born between 1980 and 1990, with follow-up until the year they turned 30; an age after which, according to prior research, it is uncommon to receive a first criminal conviction (see for example Skardhamar, 2014; von Hofer, 2014). We used the first sanction type of each kind as an index sanction and analyzed the preceding offending trajectories and background characteristics. As offenders at the severe end of the penal system are often recidivists, we focused on offenders receiving the first sanction type of each kind.

The purpose of this study is to provide knowledge about recorded offending trajectories preceding index sanctions of different types. This information is valuable for criminal policy, since it identifies potential points at which criminal behavior can be addressed before it leads to offenses resulting in more severe sanction types. We provide an overview of the crime careers preceding different sanction types, thus describing both the populations and sentencing practices on different steps of the punishment ladder.

## Data and Methods

The data used in this study were based on register datasets from various administrative sources accessible via a remote access system provided by Statistics Finland (permission number TK/2575/07.03.00/2025). By using an encrypted personal identification code we linked individuals’ sociodemographic information to conviction data, including birth year and sex. The full study population consisted of 365,540 men and 349,067 women born between 1980 and 1990, who were alive and living permanently in Finland at the age of 14. This was used as the baseline for estimating the proportions of population who would receive criminal sanction, further divided into subsamples suitable for analysis of the index sanctions. The cohorts were chosen so that community service was available in full for every cohort since the beginning of the age of criminal responsibility (15), with an equal follow-up time until 30 dictated by the availability of the data, ending in 2020. Restricting the sample to those who were in the population at age 14 excludes individuals who immigrated later, as well as individuals who emigrated or deceased before this.

Our main dataset consisted of all criminal convictions from district courts. One conviction can include multiple crimes, which are categorized into a principal offense (the most severe crime according to the Finnish penal code), secondary offense and other offenses. We identified the first sanction type of each kind, resulting in data suitable for analysis of the proportion of individuals who had received these punishments before the age of 30. The proportion of persons sentenced to different types of sanctions was estimated by creating a yearly dummy (0/1) for every man and woman born between 1980 and 1990, indicating whether they had received the sanction type in question. We used logistic regression and calculated predictive margins, which show the cumulative proportion of individuals who had received each sanction type by the age of 30.

To further investigate the criminal histories preceding index sanctions, we created subsamples for suspended imprisonment (*n* = 41,942), community service (*n* = 7,040) and imprisonment (*n* = 6,518). For these subsamples, we provide descriptives of the index sanction (Table 1) and criminal histories preceding the index sanction (Table 2). To estimate the number of recorded crimes preceding index sanctions, we used data that include all crimes from sentences. Unlike a prior study that counted similar incidents within a sentence as a single case based on the most serious offense (Farrington et al., 2023), we included all crimes from all sentences in order to better capture the full scope of preceding crimes among the convicted individuals. We also included minor fines imposed by the police and prosecutors to obtain official criminal histories that were as comprehensive as possible. Each offense has a code that refers to the specific crime in the Criminal Code, which was used to categorize crimes into five groups. The offenses were categorized as violent (including sex offenses), property, traffic, drug-related, and other crimes. The least serious traffic infractions were removed, as there have been significant changes in their control measures during the follow-up period (Pitkänen et al., 2022). We also excluded conscientious objectors (*n* = 347), as persons sentenced to imprisonment for refusing mandatory military service can be seen as a distinct group among individuals receiving criminal convictions.

The conviction data were structured in two different ways in order to track back from the index sanction. First, we looked at the order of sanctions (Figs. 2a-c). The order of the sanction preceding the index sanction is t = -1, the sanction before that is t = -2, and so forth. The sanctions were organized by the date on which the judgement was pronounced. This enabled us to examine the types and quantities of sanction types preceding the different steps on the ladder of punishment. Our analysis does not cover the timing of earlier sentences relative to the index sanction, but describes the number and distribution of preceding sanction types.

Second, as is typical in research on crime trajectories, we used calendar time and measured offending at equally spaced intervals. We used generalized additive models (GAM) to estimate the predicted number of recorded crimes before the index sanction in each of the three subsamples. The data were organized by calculating the temporal distance between the offense dates preceding the index sanction and the date of the principal offense in the index sanction, followed by aggregating the counts at the quarterly level. If an individual recorded multiple offenses in the same yearly quarter, these were treated as aggregate counts. GAMs were estimated for all index sanction types (suspended imprisonment, community service and imprisonment), as well as separately by the principal offense of the index sanction used as outcome (e.g. individuals sentenced to suspended imprisonment with property crime as the principal offense).

Only crimes from sentences prior to the index sanction were included, meaning preceding crimes that resulted in a sentence after the index sanction were excluded. The estimates of recorded crimes preceding the index sanction are likely conservative, as we excluded all offenses included in the index sanction, regardless of their timing in relation to the principal offense. Since it was not possible to differentiate when authorities became aware of the crimes included in the index sanction, it was more reliable to include only crimes in the preceding sentences. The resulting predictions can be interpreted as the officially recorded criminal career preceding the index sanction.

We present the GAMs as trend models, with time relative to index offense as the independent variable and the number of crimes recorded as the dependent variable (Figs. 3a-c). The time points are divided into quarters of years, producing estimates of the recorded crimes in yearly quarters before the index sanction. We used the gam-function of the *mgcv* package in R (Wood, 2011) to fit generalized additive models using thin plate regression splines (bs = “tp”, k = 10), with smoothness estimated using restricted maximum likelihood (REML) and assuming a Poisson distribution. These model specifications were assessed suitable based on Wood et al. (2016). The GAM formula ensures that the potential non-linear relationship between time and number of crimes recorded is maintained. As our aim is to examine the temporal relationship between the number of crimes recorded and the timely distance from the principal offense in the index sanction, GAMs are suited to detect potential fluctuation and due to the smoothing penalties reducing overfitting. We used the *gam.check* function from the *mgcv* package (Wood, 2011) to evaluate the suitability of the model specifications, and the diagnostics indicated that the chosen specifications were appropriate for our purposes (see supplementary materials for gam.check results).

## Results

**Fig. 1:**
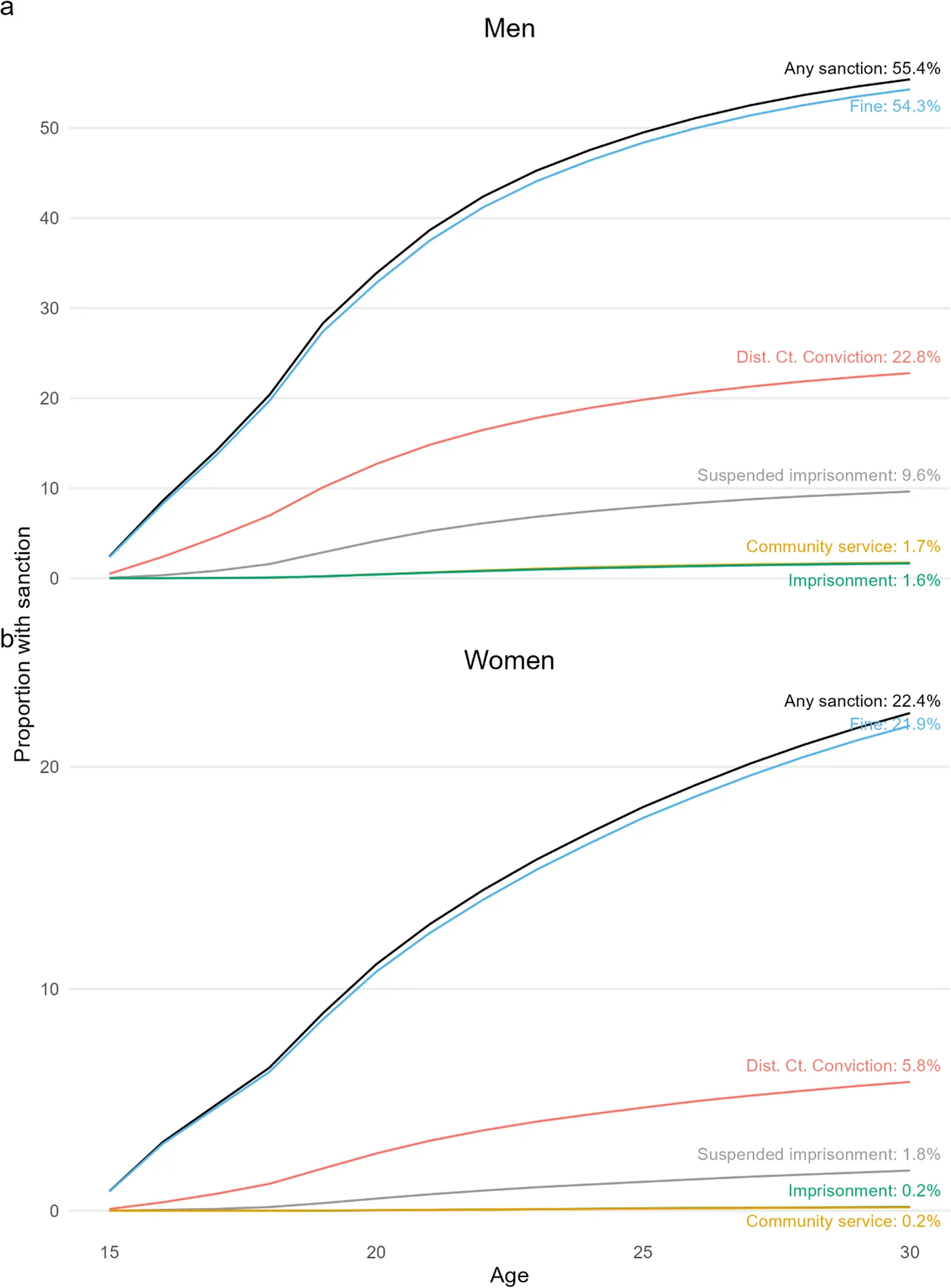
**a** and **b**. Proportion of men and women born in 1980–1990 sentenced to different sanction types by the age of 30. The categories “Any Sanction” and “Fine” include minor fines issued by the police and prosecutors

Figure 1a shows the predictive margins of the cumulative proportion of men sentenced at least once to each sanction type by the age of 30. Of men born in 1980–1990, over half (55.4%) had been issued a minor fine or convicted at least once before the age of 30 (see supplementary materials for CIs). The most common sanction was fines (54.3%), the least severe sanction type. When excluding minor fines issued by the police and prosecutors, 22.8% of men had received a conviction in district court. This category includes fines issued by the court, suspended imprisonment, community service and imprisonment. When looking at criminal convictions more severe than fines, 9.6% of individuals were sentenced to suspended imprisonment. Community service and imprisonment were less common, with 1.7% and 1.6% of men receiving these respectively.

Women received fewer sentences, with 22.4% being issued a minor fine or convicted before the age of 30 (Fig. 1b). Similar to men, the most common sanction was fines (21.9%), while 5.8% was convicted in district court. Suspended imprisonment was imposed on 1.8% of women, a proportion similar to that of community service and imprisonment in men; these sanction types concerned 0.2% of women.^1^

When examining index sanctions of each kind in more detail, suspended imprisonment was the offender’s first entry in their criminal history in 23.6% of cases (Table 1). Community service and imprisonment were less commonly the first sanction an individual had received; for 0.4% and 2.2% of offenders respectively, it was their first sanction. The median age of receiving a first suspended imprisonment (21 yrs) was lower compared to community service (23 yrs) and imprisonment (23 yrs). While the difference in age was small, the difference in average amounts of previous sanctions and crimes recorded was notable. Individuals receiving their first suspended imprisonment had a median of 2 other previous sanctions, while first-time receivers of community service had received a median of 9 other sanctions and persons sentenced to imprisonment 13 other sanctions. Crimes related to substances (i.e., driving under influence, drug use offenses, crimes related to illegal distribution of substances) recorded before the index sanction were common among individuals receiving community service (82.3%) and imprisonment (78.4%).

Regarding the crime types resulting in index sanctions, traffic crimes accounted for almost half (47.4%) of suspended imprisonments imposed on offenders receiving this sanction type for the first time, with community service showing a similar proportion. Overall, the crime types that led to non-custodial index sanctions were similar in both sanction types. However, imprisonment was clearly different: traffic crimes accounted for 20.5% of first-time imprisonments, whereas they accounted for almost half of first-time sentences in non-custodial sanction types. Compared to non-custodial sentences, violent and drug-related crimes were more common as the crime types resulting in imprisonment. A possible explanation for the higher proportion of first-time offenders receiving imprisonment for these types of crimes is that serious violent and drug-related offenses can lead to sentences of imprisonment even when the offender has no previous criminal history. In particular, the proportion of violent crimes resulting in an index sanction was higher among those sentenced to imprisonment (41.6%) than those receiving their first suspended imprisonment (22.5%) or community service (21.3%).

**Table 1:** Descriptives of index sanction by sanction type. Minor fines are included

	Suspended imprisonment (*n* = 41,942)	Community service (*n* = 7,040)	Imprisonment (*n* = 6,518)
Women	14.9%	7.5%	9.0%
Immigrant background^2^	4.1%	3.3%	5.7%
Age			
Median	21	23	23
Mean	22.0	23.2	23.4
First sentence	23.6%	0.4%	2.2%
Crimes in index sanction			
Median	2	2	3
Mean	2.7	3.6	5.6
No. of prior sanctions			
Median	2	9	13
Mean	3.4	12.9	16.4
Substance-related crime before index sanction	26.3%	82.3%	78.4%
Sentence length (days)			
Median	60	90	255
IQR	65	75	575
Mean	100	102	517
Deceased before 30	3.5%	5.6%	6.2%
Principal offense of the sentence			
Violent	22.5%	21.3%	41.6%
Property	16.7%	21.6%	20.2%
Traffic	47.4%	46.8%	20.5%
Drug	7.2%	4.8%	11.5%
Other	6.2%	5.5%	6.2%

^2^Immigrant background was defined as having both parents, or the only known parent, being foreign-born. The share of individuals in the entire study population (N = 714,607) is 2.0%

**Fig. 2:**
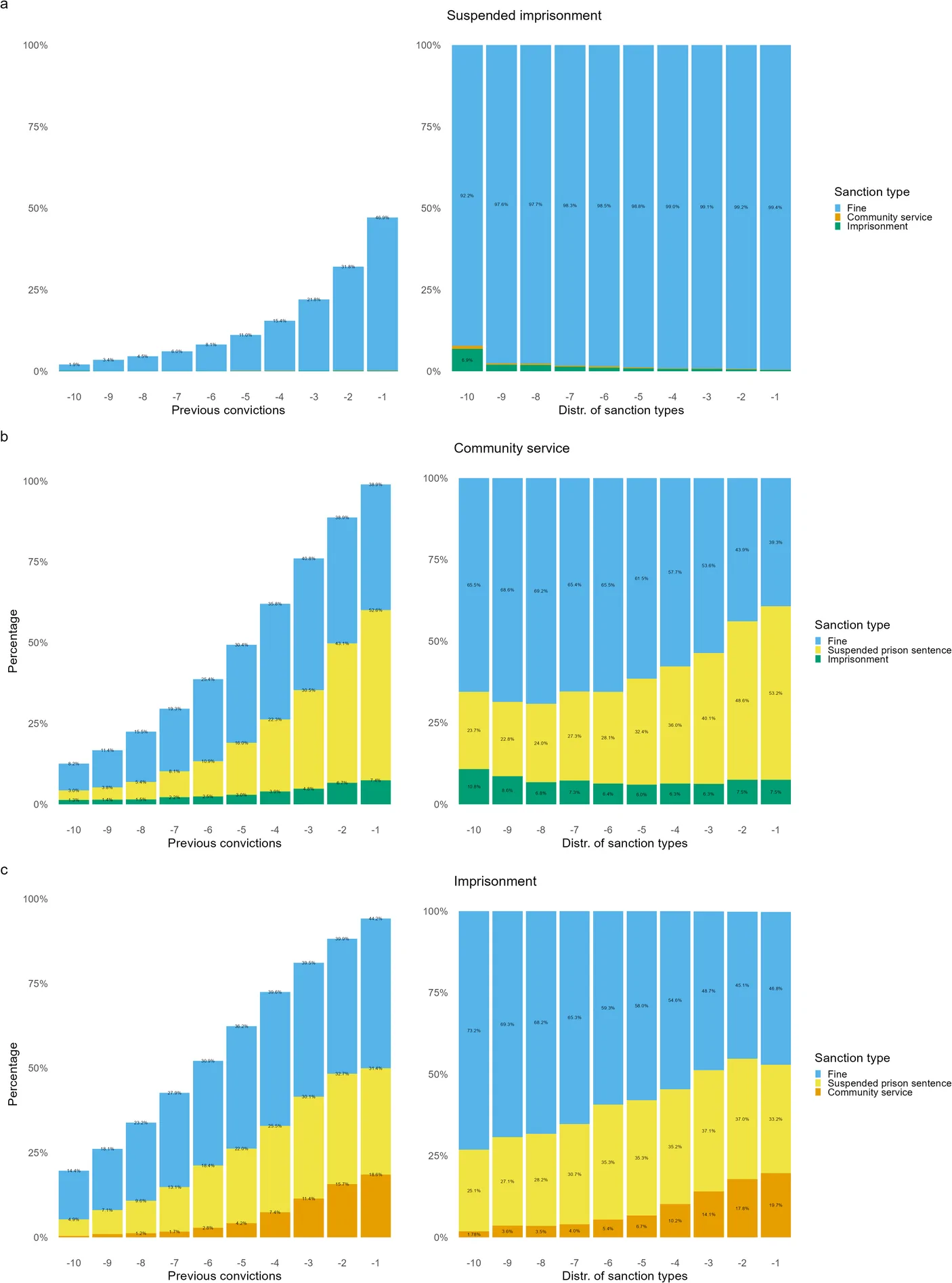
**a**, **b** and **c**. Distributions of sanction types preceding the index sanction among first-time recipients of each index sanction. Left panel shows the percentage of individuals with previous sanctions, and the right panel shows the distribution of sanction types within those populations. Minor fines have been excluded, see Appendix 4a-c for plots with minor fines

**Fig. 3:**
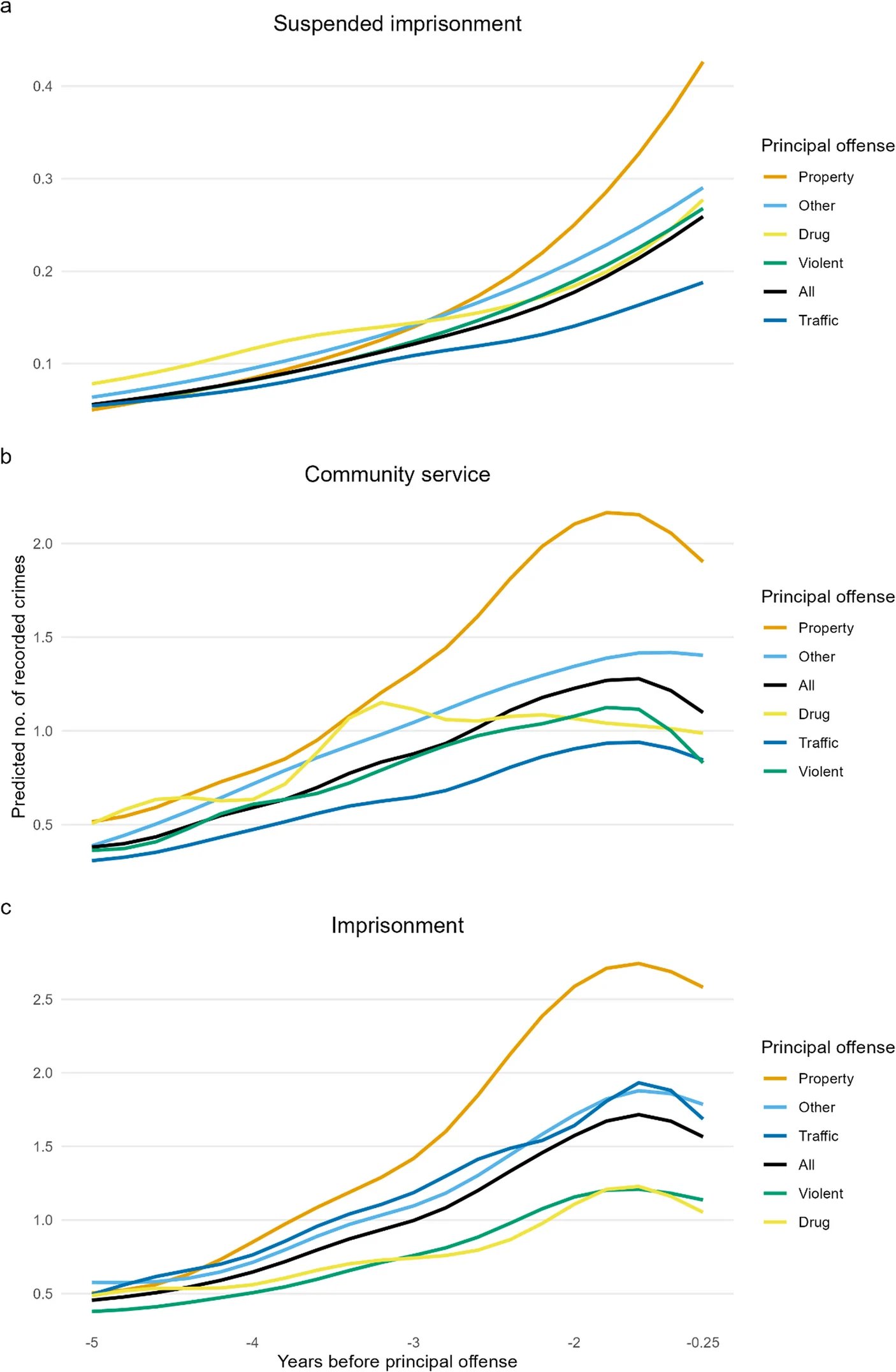
**a**, **b** and **c**. Predicted number of recorded crimes preceding index sanction by sanction type and principal offense. Includes all crimes extracted from sanctions preceding the index sanction

In the following descriptives (Table 2) and analyses (Figs. 2a-c and 3a-c) we concentrate on the subpopulation of offenders with criminal convictions preceding the index sanction (e.g., the 76% of persons sentenced to suspended imprisonment with previous convictions). Offenders with prior criminal history received their first conviction on average at age 17, showing similar onset age across sanction types. This was expected, since persons receiving the most severe punishments have typically also received the less severe punishments, resulting in an inevitable overlap in offender subpopulations.

While suspended imprisonment was the first sanction imposed on individuals in a quarter of cases involving suspended imprisonment, the 76% of offenders sanctioned to suspended imprisonment with a criminal background had received a median of three prior sanctions. Offenders receiving their first community service had a more severe offending history, with a median of 9 former convictions and a median of 15 individual crimes included in the former sentences. The number of sanctions and crimes was again higher when receiving the first imprisonment, with a median of 13 former sentences and 23 individual crimes.

First-time recipients of imprisonment were convicted of property crimes as their first official crime (32.4%) more often than first-time recipients of suspended imprisonment (24.2%) and community service (27.2%). Traffic crimes were less common as the first crime among individuals sentenced to imprisonment when compared to first-time recipients of non-custodial sentences. The somewhat small differences (as in the case of onset age) were largely explained by the fact that offenders receiving severe sanction types had also been sentenced to lesser sanctions before. Considering this, the differences in property and traffic crimes can be taken as noteworthy.

**Table 2:** Descriptives of criminal background by index sanction type among offenders with criminal convictions preceding the index sanction. Minor fines are included

	Suspended imprisonment (*n* = 32,051)	Community service (*n* = 7,011)	Imprisonment (*n* = 6,374)
No. of previous sanctions			
Median	3	9	13
Mean	4.5	12.9	16.8
No. of previous crimes			
Median	3	15	23
Mean	5.4	23.6	29.3
Substance-related crime before index sanction	34.4%	82.6%	80.2%
Age at first offense			
Median	17	17	17
Mean	18.0	17.4	17.2
First offense crime type			
Violent	6.1%	8.7%	9.2%
Property	24.2%	27.2%	32.4%
Traffic	43.9%	40.3%	33.4%
Drug-related	3.9%	3.0%	4.0%
Other	22.0%	20.8%	21.1%

The more detailed analyses of sanction trajectories (Figs. 2a-c) show the proportions of individuals with different numbers of previous sanctions and the sanction types these previous convictions consist of (see Appendix 1 for aggregate-level proportions of preceding sanctions). The right bar chart shows the distributions of sanction types by conviction order (i.e., first conviction, second conviction, and so on), while the chart on the left shows the proportions with previous convictions by number of convictions and sanction type. As offenders convicted to community service or imprisonment possess extensive criminal histories with numerous minor fines issued by the police or prosecutors, we have excluded minor fines from these. The plots including minor fines are shown in Appendix 2, with over 50% (community service) and 75% (imprisonment) having received ten or more sanctions if minor fines are included.

In 47.2% of cases, offenders receiving their first suspended imprisonment had received at least one former conviction in district court (left chart). Furthermore, 32.1% had two or more former convictions, which again decreases to 22.0% with three or more convictions. Almost all of these prior convictions were fines (left chart). In some rare cases, offenders had been sentenced to imprisonment before receiving their first suspended imprisonment.

Sanction types prior to the index sanction became more severe as the index sanction approached. Suspended imprisonment was overwhelmingly preceded by fines, whereas community service and imprisonment were preceded by a more mixed history of sanctions. The proportion of suspended imprisonments was larger in the sentences immediately before community service and imprisonment, but fines were more common in the earlier sentences. The preceding sanction trajectories indicate that the punishment ladder is ascended somewhat gradually, with individuals moving from lesser sentences to more severe ones. The difference in preceding sanctions between community service and imprisonment is noteworthy, as community service preceding imprisonment is more common than imprisonment preceding community service.

Individuals sentenced to their first community service had more severe and varied criminal histories, with almost all having at least one former sentence, 88.8% having two or more and 76.0% having three or more sentences. 12.5% of the offenders had received 10 or more individual sanctions before their first community service, indicating that a significant proportion of offenders had extensive criminal histories. Among those with several prior convictions, fines constituted a larger proportion of the overall sanction type distribution, and suspended imprisonment was more common when moving closer to the index sanction.

Sentences received before first imprisonment showed a similar trend to community service: 94.2% of the individuals had at least one former conviction and 19.7% had at least 10 prior convictions. When looking at the distribution of sanction types, the proportion of suspended imprisonment and community service increased when moving closer to the index sanction, while the proportion of fines decreased. In general, the results show that, regarding community service and imprisonment, the closer the previous convictions are to the index sanction, the more common and severe the prior sanction types are. Regarding suspended imprisonment, only the number of prior convictions seems to increase when approaching the index sanction.

In Figs. 3a-c we show the predicted number of crimes recorded before the principal offense in the index sanction (see supplementary materials for tables with CIs). The predicted crimes are shown by the crime type of the index crime; e.g., separate estimations for individuals whose index crime was property crime, violent crime, and so on. The analyses show that the predicted number of recorded crimes increased before the first suspended imprisonment (Fig. 3a). Offenders convicted of property crimes had the largest predicted number of offenses prior to their first suspended imprisonment. Criminal records preceding suspended imprisonment for traffic crime offenses were the most modest, with a peak number of 0.19 predicted number of recorded crimes in the quarter before the principal crime of index sanction.

As could be predicted from the sentence histories presented previously, offenders sentenced to community service showed higher predicted number of recorded crimes preceding the index crime, with 1.1 predicted number of recorded crimes in the preceding quarter. The predicted number of recorded crimes was highest among offenders convicted of property offenses, with predicted number of 1.9 crimes recorded in the preceding quarter. As in suspended imprisonments, individuals sentenced for traffic-related offenses showed a low number of predicted crimes in the quarter preceding the index offense (0.8).

Offenders sentenced to imprisonment had the highest predicted number of recorded crimes in the preceding quarter (1.6), and individuals convicted of property crimes again had the most extensive offending trajectory (predicted number of 2.6 crimes in the preceding quarter). Contrary to suspended imprisonment and community service, which had traffic index sanctions associated with a low number of predicted crimes, those sentenced to imprisonment for traffic crimes had the third-highest level of offending. This is likely due to traffic crimes rarely being the principal offense in imprisonment sentences (see Table 1), indicating that a more extensive criminal history is required for the offense to lead to imprisonment.

The figures for community service and imprisonment show a decline in predicted number of recorded crimes preceding the principal offense in the index sentence. This is likely because the crimes included in the index sentence were excluded in the analysis, as crimes that occurred close to the principal crime in the index sentence are probably handled in the same trial and sentence. We conducted additional analyses with crimes included in the index sentence but preceding the principal offense of the sentence, which softened or removed the decline (Appendix 5a-c).

As the pre-age-15 yearly quarters were coded as zero recorded crimes in the GAM models, we ran additional analyses for individuals who received their index sanction after the age of 20. This did not meaningfully alter the results reported in main analysis (see supplementary materials for results).

## Discussion

Receiving any criminal sanction was common in the birth cohorts studied, with 55% of men and 20% of women being issued a minor fine by the police or prosecutor or convicted in court before the age of 30. If excluding minor fines, a court-ordered sentence was imposed upon approximately 23% of men and 6% of women. In the Nordic context, the conviction rates presented here are somewhat similar to Sweden (Sivertsson et al., 2021), while Norway has shown lower rates in a study conducted with synthetic cohort approach (Skardhamar, 2014). While the proportion of individuals receiving any criminal sanction was high, the proportion with a sanction other than a fine was notably lower.

There were notable overlaps in the populations examined, since the offenders receiving the most severe punishments had already had multiple contacts with the criminal justice system. However, these thresholds for receiving different sanction types provide important insights for criminal career research. The focus on first-time recipients of sanction types highlights that these individuals have already had numerous criminal justice system contacts before receiving the more severe sanctions. This indicates that while the prison population, for example, consists mainly of individuals who have already served previous prison sentences, majority of individuals sentenced to prison for the first time are persistent offenders with multiple less severe sentences on their record.

The analyses of offending trajectories preceding index sanctions showed that, as expected, the predicted number of recorded crimeswas higher for the more severe sanction types. There was a clear increasing trend in offending trajectories in the five years preceding the offense that led to the index sanction, with persons convicted of property offenses showing the highest predicted number of recorded crimes in the quarter preceding the index offense. For a property crime to result in community service or imprisonment, the offender’s criminal career is probably required to be more extensive than in e.g., violent crimes.

Since there are no official sentencing guidelines concerning previous offending as an aggravating factor when determining a sentence in Finland, this study provides information regarding the judicial system and the ladder of punishment model. It should be noted that it is unclear how prosecutors and judges evaluate preceding offenses and sanctions when an individual is accused of a crime, and, as mentioned, they employ independent discretion in the imposition of sentences. However, when analyzed as aggregate data, the criminal records preceding sanctions more severe than fines were extensive. In the cases of community service and imprisonment, it is notable that 12% and 19% respectively had received 10 or more prior convictions before receiving the sanction type in question for the first time. Suspended imprisonment is more often the base sanction for entering the criminal justice system, and yet 76% had been issued with at least one court-ordered fine before receiving this sanction, with 9% having received 10 or more fines.

Although suspended imprisonment is largely preceded by fines, the threshold for receiving a suspended imprisonment appears to be relatively low when compared to harsher sentences. Offenders remain on the same step of the punishment ladder for a long time before moving from suspended imprisonment to community service or imprisonment. This gap between suspended imprisonment and more punitive sanction types should be addressed when evaluating the functioning of the criminal justice system. Since the harsher sanctions are typically preceded by multiple suspended imprisonments and fines, these could potentially be moments for implementing interventive measures.

Substance-related offenses (e.g., DUIs and drug-related offenses) were common in the criminal histories of all sanction types examined. The proportion of first-time recipients of community service with preceding substance-related crimes was notably high (82%), as one of the common reasons for deciding against community service is problems with substance abuse. The extensive criminal careers combined with common substance-related crimes that precede both community service and imprisonment may be seen as indicators of serious disadvantage. This could partly explain the positive health and mental health effects of imprisonment with rehabilitative measures in the Nordic context (Bhuller et al., 2025; Hjalmarsson & Lindquist, 2022). Since the population experiencing incarceration for the first time is already in such an unfavorable position, it is expected that compensatory interventions produce a positive effect. The same reasoning could also partly explain why disadvantages persist beyond prison sentences (Aaltonen et al., 2017).

### Limitations

The high-quality register data used in this study provide adequate conditions for analyses, but there are limitations that should be acknowledged when interpreting the results. Some of the entries in the register data are unlikely to be accurate, since the officials responsible for the entries might not have had exact information when producing the records. For example, this problem can be seen in register entries with regard to the dates upon which crimes were committed. In sentences involving multiple crimes that have unclear, imprecise or long-lasting timing, the same date of commission may be registered for all crimes. This may have produced a bias in the predicted amounts of crime in the GAM models, since a very large number of crimes may have been registered on the same day when in reality the crimes occurred over a longer period of time. However, the additional analyses in which we excluded crimes of the same type recorded on the same day produced similar results as those shown in Figs. 3a-c (see supplementary materials).

While register data have several advantages when compared to self-reported data (Hovde Lyngstad & Skardhamar, 2011), the data lack information on early criminal behavior. Since the beginning of each offender’s official criminal history is bound to the age of criminal accountability, conviction data have no information on criminal behavior before the age of 15. However, since offenses committed before the age of 15 are not considered in sentences received after turning 15, these offenses should not have any influence on sentencing practices. The officially recorded criminal histories are thus reliably presented since crimes before the age of 15 should not have influence on any sanction types received for the first time.

Another point to consider is that although first convictions become less common after the age of 30, they do occur. Restricting the data to age 30 therefore excludes individuals convicted to different sanction types at older ages. These individuals may constitute a distinct group, as, for example, the interval between convictions may be longer when compared to individuals receiving first convictions before the age of 30. The interpretations in this study regarding repeated criminal justice contacts preceding index sanctions and potential intervention points may therefore not apply to individuals who receive their first such sanction at an older age.

Furthermore, we do not have information on appeals. It has been estimated that in approximately 10% of district court decisions considering crimes, one of the parties appeals the judgement to a higher court (Aaltonen, 2019). Although some of the decisions may be altered in a higher court, we consider it unlikely that this would change the overall picture presented in our study.

It should also be noted that the Finnish criminal justice system recognizes several other sanction types, i.e., monitoring sentences, juvenile punishment, and suspended imprisonment with supervision. While it is possible that these sanction types are used as intermediate steps on the punishment ladder, and that the analyses we provide therefore contain some inaccuracies, the use of these sanctions has been marginal since their implementation (see: Halén et al., 2024; Kuusisto Mäkelä, 2023; Lähteenmäki-Meriluoto, 2024). We argue that our results would not be greatly affected if we included these sanction types, since they have not been utilized widely and would only concern a limited number of individuals.

### Conclusions

Our study on the sanction and offending trajectories preceding first non-custodial and custodial sentences produced novel information about the populations receiving these punishments. The thresholds for receiving different sanction types do not increase gradually, as the gap in criminal histories when comparing suspended imprisonment to more severe sanction types is large. While not a direct test of proportionality in terms of aggravation on condition of previous offending, our study shows that the criminal histories preceding sanction types more severe than conditional imprisonment are typically extensive. As for the ladder of punishments model, the step to suspended imprisonment is relatively low when compared to the subsequent steps to more severe sanction types. Offenders sentenced to community service or imprisonment have typically accumulated multiple lesser sanctions, and individuals with multiple fines or suspended imprisonments could be identified as potential targets for interventive measures.

Since a high proportion of offenders sentenced to community service and imprisonment have a preceding offense related to substances, alcohol and drug rehabilitation could be addressed during contacts with the criminal justice system. Further studies are necessary to better identify these potential situations and what type of support could reduce later escalation for individuals with repeated minor sanctions. For example, the Finnish Criminal Law states that suspended imprisonment can be imposed with additional monitoring, which could include interventive measures and tasks that target prevention of further crime (Criminal Code of Finland Chap. 6, Sect.  10). However, this is very rarely utilized (Kuusisto Mäkelä, 2023).

Finally, as trials and the decisions made by courts are, with some exceptions, public by law (Act on the Publicity of Court Proceedings in General Courts, 370/2007), the overall assessment of sanctions and the official crime careers leading to them can be seen as part of this publicity principle. The publicity principle has been argued to be an important part of both due process and public trust in the criminal justice system (Jokela, 2008). Our results complement these aspects by providing additional means for evaluating the system.

## Supplementary Information

Below is the link to the electronic supplementary material.

Supplementary Material 1 (DOCX 129 KB)

## Figures and Tables

**Fig. 4:**
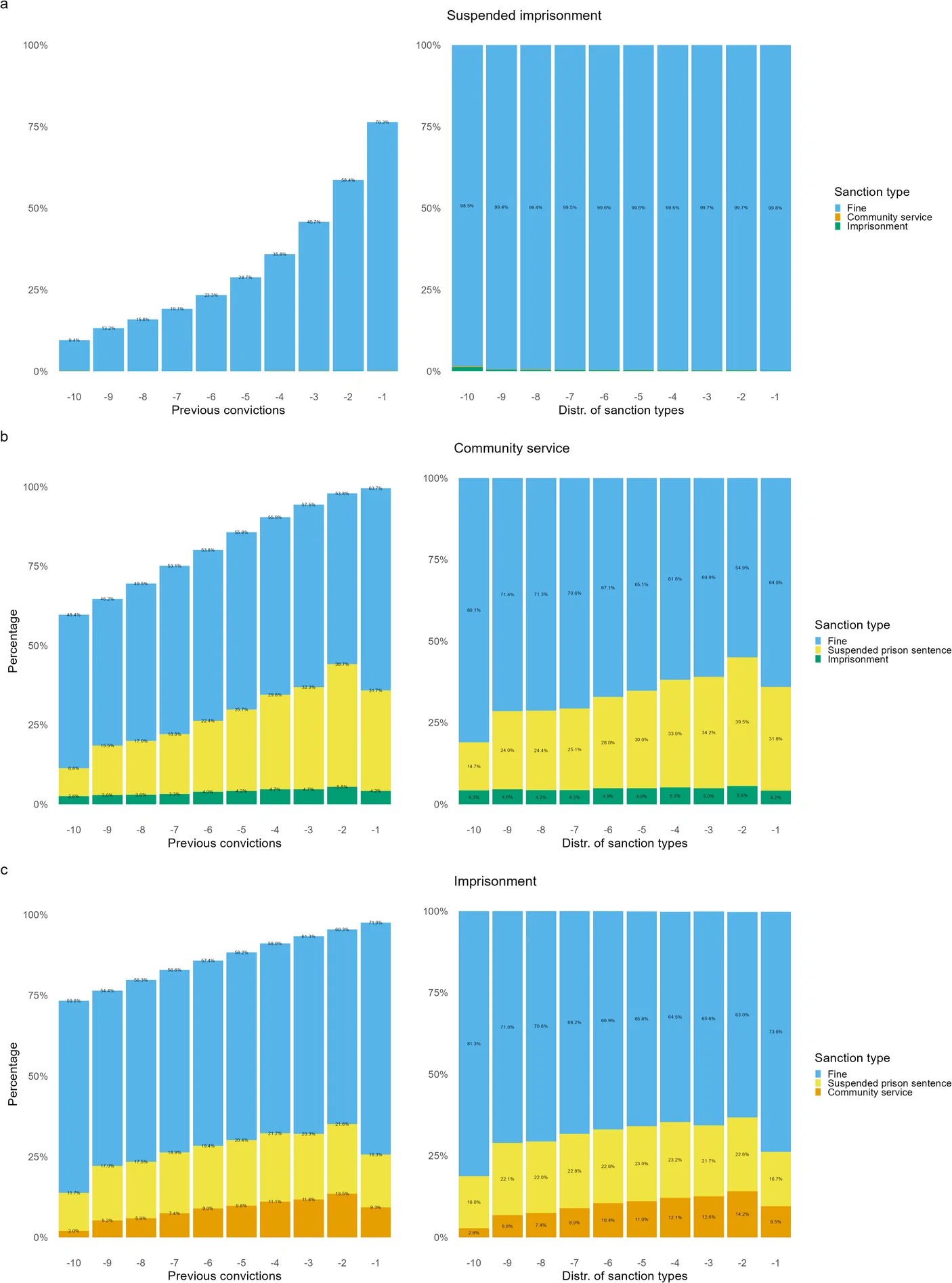
**a**, **b **and **c**. Distributions of sanction types preceding the index sanction among first-time recipients of each index sanction. Left panel shows the percentage of individuals with previous sanctions, and the right panel shows the distribution of sanction types within those populations. Includes minor fines issued by police and prosecutors

**Fig. 5:**
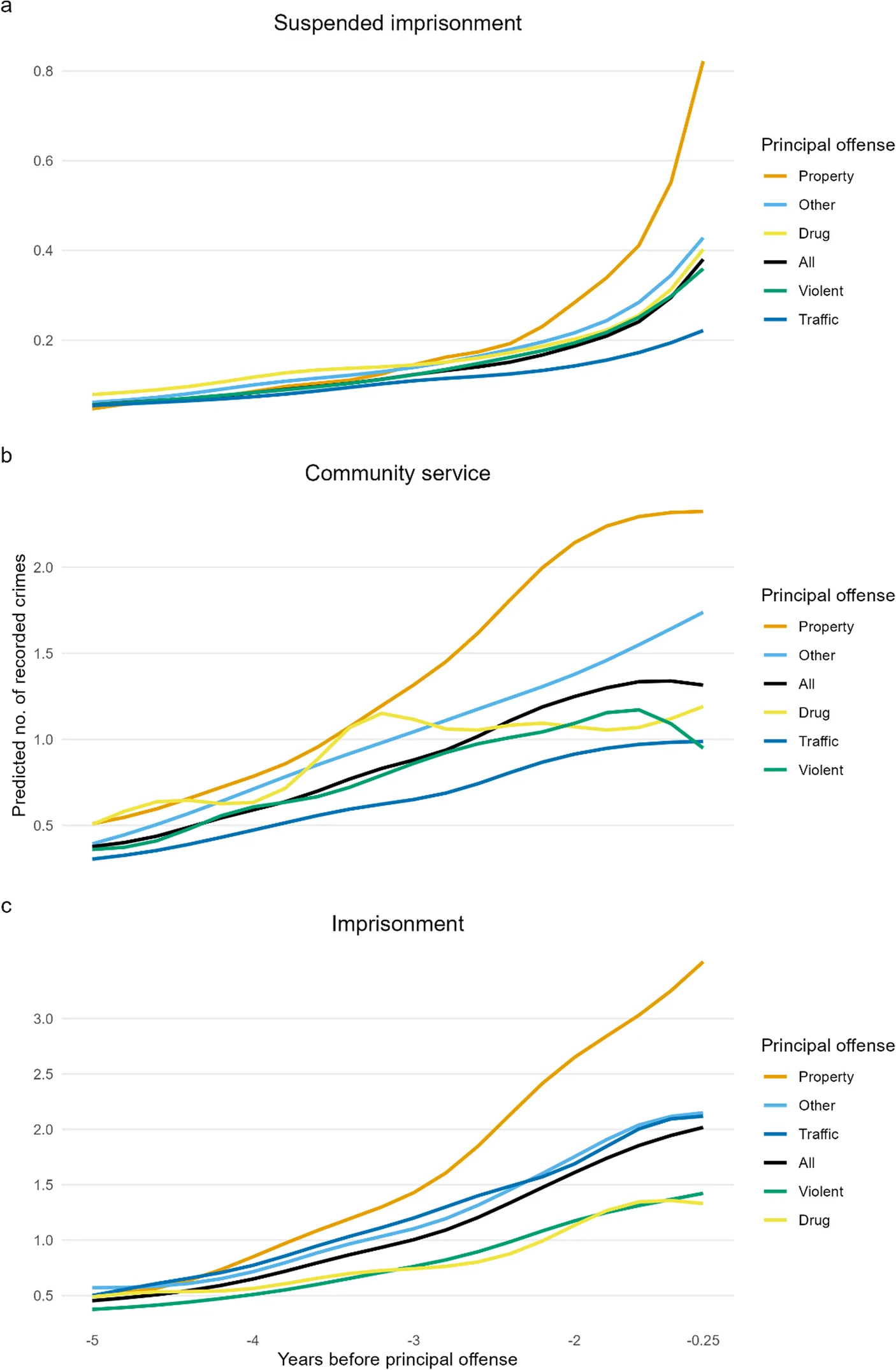
**a**, **b** and **c**. Predicted number of recorded crimes preceding index sanction by sanction type and principal offense. Includes all crimes extracted from sanctions preceding the index sanction and crimes included in the index sanction but preceding the principal offense

## Data Availability

The individual-level register data are not publicly available due to Finnish privacy laws and data protection regulations. Access to the data may be obtained by applying for permission from Statistics Finland.

## Appendix

**Table 3:** Proportion of individuals previously sentenced to different sanctions by index sanction

	Fine	Suspended imprisonment	Community service	Imprisonment
Suspended imprisonment (*n* = 42,200)	76.4%	*	0.1%	0.4%
Community service (*n* = 7,040)	95.8%	96.8%	*	15.3%
Imprisonment (*n* = 6,518)	96.7%	81.1%	35.4%	*

## Footnotes

1 Kaplan-Meier analysis was conducted as a sensitivity check for suspended imprisonment, community service and imprisonment with censoring for death, and the results showed no significant change compared to our models (available by request).
