# Supplementary material for: Recorded Offending Trajectories Preceding Different Sanction Types: A Register-Based Study on the Ladder of Punishments
Source: J Dev Life Course Criminol. 2026 Oct 15;12(1):35. doi: 10.1007/s40865-026-00326-8 (PMC13672512; doi:10.1007/s40865-026-00326-8)
Supplement: Supplementary file 1 [file 40865_2026_326_MOESM1_ESM.docx]

Supplementary materials

Table 1a & 1b. Predictive margins of men and women born in 1980-1990 sentenced to different sanction types by the age of 30. The categories “Any Sanction” and “Fine” include minor fines issued by the police and prosecutors.

1a. Predictive margins for men (smallest values removed due to data anonymity guidelines).

1b. Predictive margins for women (smallest values removed due to data anonymity guidelines).

Tables 2a, 2b & 3c. GAM results from main analyses (Figures 3a, 3b & 3c in the study) with gam.check results for “all” category..

2a. Suspended imprisonment

2b. Community service

2c. Imprisonment

Tables 3a, 3b & 3c. GAM results from analysis with crimes in index sanction included.

3a. Suspended imprisonment

3b. Community service

3c. Imprisonment

Tables 4a, 4b & 4c. GAM results from analysis with individuals who received their index sanction at age >= 20.

4a. Suspended imprisonment

4b. Community service

4c. Imprisonment

Tables 5a, 5b & 5c. Results from GAMs with crimes of same type committed on same day excluded.

5a. Suspended imprisonment

5b. Community service

5c. Imprisonment
